# An examination of the workflow process of the screening, brief intervention, and referral to treatment program

**DOI:** 10.1186/1940-0640-10-S1-A25

**Published:** 2015-02-20

**Authors:** David Kaiser, Georgia Karuntzos, Carol Council

**Affiliations:** 1 Behavioral Health Economics Program, RTI International, Research Triangle Park, NC, 27709-2194, USA

## Background

Substance abuse and dependence are widely recognized as widespread societal problems, and most people who engage in risky substance use do not recognize it as a problem. The Substance Abuse and Mental Health Services Administration (SAMHSA) launched the Screening, Brief Intervention, and Referral to Treatment (SBIRT) grant program in 2003 to identify, reduce, and prevent problematic use, abuse, and dependence on alcohol and illicit drugs among individuals who would not typically seek treatment. Following the evidence base, SBIRT programs were implemented in general health-care settings, with the aim of integrating behavioral health services in locations that provide an opportunity to identify risky patients and provide them with an appropriate level of treatment.

In 2008, SAMHSA sponsored an evaluation of the SBIRT program among its third cohort of grantees. A key focus of the evaluation was to understand the SBIRT workflow process, or the process by which clients interact with health-care personnel to obtain SBIRT services. SBIRT has been implemented in many treatment settings, and each setting has adjusted the workflow process to meet its respective needs. This study focuses on the overall workflow process, adaptations, and key observations in emergency departments and ambulatory clinics.

## Methods

A descriptive and qualitative analysis was conducted to construct workflow charts across treatment setting to depict the most common process observed. The primary data source was observations of 59 SBIRT practitioners across 21 treatment settings. The observations provided two important components: the recorded steps of the observed workflow process, and delivery times of SBIRT services. Supplemental data were also taken from three other sources to gain a deeper understanding on workflow: qualitative data from 170 different interviews with stakeholders, program administrators, practitioners, and local evaluators provided information about workflow in different settings; a review of original and updated official grantee documents to track changes in workflow over time; and estimates of the total number of patients receiving SBIRT services from the Government Performance and Results Act.

## Results

Generally speaking, the workflow process did not vary substantially across settings; existing clinical processes, health information technology, and patient characteristics drive subtle differences in workflow. Co-location of SBIRT providers and integration with electronic health records lead to efficient service delivery patterns. Interruptions of SBIRT service delivery by health-care providers, patient variation and length of stay, and the veracity of substance abuse reporting by patients pose challenges to a stable workflow. Incentive programs to help integrate the SBIRT into settings appear to be effective.

## Conclusions

SBIRT workflow is being adapted to efficiently operate within medical settings, illustrating that the integration of behavioral health and medical care services is being performed successfully. The presented workflow diagrams and described variations have implications for health administrators and treatment providers on the ways to implement, manage, and model an SBIRT program to fit within any medical setting. Provisions within the Patient Protection and Affordable Care Act of 2010 call for the integration of behavioral health and medical care services, and the SBIRT workflow demonstrates potential of successful integration.

